# The gut microbiota induces melanin deposits that act as substrates for *fimA*-mediated aggregation of *Salmonella* Typhimurium and enhance infection of the German cockroach vector

**DOI:** 10.1128/spectrum.02119-23

**Published:** 2023-08-22

**Authors:** Matthew Turner, Landen Van Hulzen, Jose E. Pietri

**Affiliations:** 1 Division of Basic Biomedical Sciences, Sanford School of Medicine, University of South Dakota, Vermillion, South Dakota, USA; University of Valencia, Paterna, Valencia, Spain

**Keywords:** *Salmonella*, cockroach, vector, infection, aggregation, microbiota, melanization, *fimA*

## Abstract

When *Salmonella* Typhimurium is ingested by German cockroaches, the bacteria replicate in the gut and persist for at least 7 d, enabling transmission in the feces. However, the mechanisms that facilitate survival and persistence in the cockroach gut remain poorly detailed. We previously reported the formation of biofilm-like aggregate populations of *S*. Typhimurium in the gut of cockroaches upon ingestion. We also reported that deletion of the type-1 fimbrial subunit of *S*. Typhimurium, *fimA*, leads to a reduced bacterial load in the cockroach gut. Here, we link these observations and provide further insight into the mechanism and function of *S*. Typhimurium aggregation in the gut of the cockroach. We show that *S*. Typhimurium but not *Escherichia coli* forms aggregated populations in the cockroach gut, and that aggregate formation requires *fimA* but not the biofilm formation-related genes *csgA* and *csgD*. Furthermore, we show that *S*. Typhimurium aggregates are formed using small granular deposits present in the cockroach gut, which exhibit properties consistent with melanin, as substrates. These melanin deposits are prevalent in the guts of both immature and adult cockroaches from laboratory colonies and are correlated with increased gut bacterial density while being entirely absent in gnotobiotic cockroaches reared without exposure to environmental bacteria, indicating they are induced as a response to the gut microbiota. When cockroaches lacking melanin deposits in the gut are fed *S*. Typhimurium, they exhibit lower rates of infection than those harboring melanin deposits, demonstrating that microbiota-induced melanin deposits enhance infection of the gut of the vector.

**IMPORTANCE:**

Cockroaches, including the German cockroach (*Blattella germanica*), can be both mechanical and biological vectors of pathogenic bacteria. Together, our data reveal a novel mechanism by which *S*. Typhimurium interacts with the cockroach gut and its microbiota that promotes infection of the vector. These findings exemplify the emerging but underappreciated complexity of the relationship between cockroaches and *S*. Typhimurium.

## INTRODUCTION

German cockroaches (*Blattella germanica*) are ubiquitous pests that thrive in synanthropic environments. Although their contribution to human allergic disease (i.e., asthma) is well established (1), their role in spreading infectious disease agents is underappreciated. Meta-analysis indicates that as many as 20% of German cockroaches in the field may harbor *Salmonella* spp. (2) and there are multiple reports that associate German cockroaches with outbreaks of enteric human disease, including Salmonellosis (3 – 6). Other experimental studies have demonstrated the ability of cockroaches to act as vectors of enteric pathogens via transfer on the cuticle and shedding in the feces (7 – 11). Although mechanical transmission of pathogens by cockroaches is somewhat appreciated, understanding of their potential for dynamic, biological transmission of pathogens is poorly developed. For example, when German cockroaches ingest *Salmonella enterica* serovar Typhimurium (*S*. Typhimurium), these bacteria replicate in the gut, reaching a steady state and persisting for at least 7 days without eliciting pathogenesis (11, 12). Yet, the underlying mechanisms that facilitate *S*. Typhimurium survival, persistence, and shedding in the cockroach gut are almost entirely unknown.

In addition to documenting multiple phases of *S*. Typhimurium replication in the gut of *B. germanica,* we previously reported the presence of two distinct populations of the bacteria within the gut (11). As early as 3 h after *S*. Typhimurium is ingested, biofilm-like aggregates form around melanized granular deposits present in the foregut, which we hypothesize are generated as an immune response (melanization) to other microbes present in the gut or to preexisting tissue damage (13). The aggregated populations of *S*. Typhimurium exist alongside planktonic bacteria. However, the regulation of the formation of these aggregates, including whether they represent true biofilms, as well as their importance for infection of the cockroach vector, remains undetermined.

The formation of biofilms and aggregates allows bacteria to survive harsh environmental conditions, including treatment with disinfectants and shifts in temperature, osmolarity, O_2_, CO_2_, pH, and nutrient availability (14). A variety of genes and regulatory networks are involved in biofilm and aggregate formation by *S*. Typhimurium (15). Furthermore, *S*. Typhimurium biofilms and aggregates have been shown to play important roles in colonization of both vertebrate and invertebrate hosts (16, 17).

In *Salmonella*, *csgD* is the master transcriptional regulator responsible for the switch from a planktonic to a biofilm lifestyle (15). *csgD* regulates *csgA* and *bcsA* to produce curli fibers and cellulose, respectively, which are key components of biofilms (18 – 20). These components are also co-regulated with the O-antigen of the *Salmonella* capsule to remodel the extracellular matrix for biofilm formation (21). One study showed that biofilms enhanced virulence and facilitated vertical transmission in chickens, with the genes *csgD* and *bcsA* playing critical roles (22). A separate study in chickens showed that *csgA* and *bcsA* have significant roles in biofilm formation and without them, biofilm formation as well as virulence was reduced (23). In the context of invertebrate hosts, biofilm formation by another enteric pathogen, *Vibrio cholerae*, facilitates selective colonization of the fly intestinal tract, extending the role of biofilms across distant phyla (24). The same is true for *S*. Typhimurium in *Caenorhabditis elegans*. In this invertebrate, the transcriptional regulator *SsrB* is responsible for the switch between a virulent and chronic infection state. Specifically, unphosphorylated SsrB activates *csgD*, allowing for the transcription of biofilm formation components and promoting a persistent infection (25, 26).

Relatedly, we previously showed that *S*. Typhimurium deficient in type 1 fimbriae (27) from *fimA* deletion were deficient in their ability to persist in the cockroach gut (11). However, the underlying mechanism behind this phenotype is unclear. Type 1 fimbriae are important for *Salmonella* formation of biofilms on gallstones in the gallbladders of mice and humans as a means of chronic persistence (28). Furthermore, a separate study showed that point mutations in *fimA* can affect the host tropism of *Salmonella* by altering affinity toward the ligand N-acetyl-D-glucosamine (29).

Given the above, we hypothesized that the establishment of aggregated populations of *S*. Typhimurium in the cockroach gut may involve *fimA*-mediated adhesion and serve to enhance infection of the vector. In the present study, we used a combination of microscopy, molecular biology, and bacterial culture assays to further characterize aggregate formation by *S*. Typhimurium in the cockroach gut, its underlying mechanisms, and its effects on vector infection.

## RESULTS

### Melanized deposits are common in the cockroach gut and act as substrates for *S*. Typhimurium aggregation during infection

**Fig 1:**
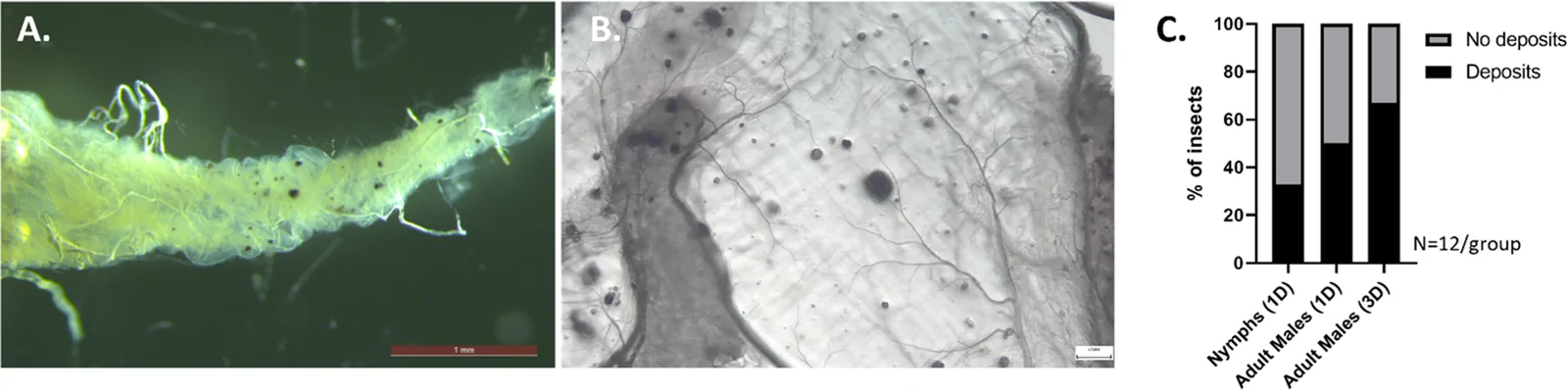
Melanized deposits are common in the gut of *B. germanica* maintained in the laboratory. (**A**) Stereomicroscopy of an uninfected gut with melanized deposits. Scale bar = 1 mm. (**B**) Compound brightfield microscopy of an uninfected gut with melanized deposits. Scale bar = 150 µm. (**C**) Prevalence of melanized deposits in the gut of uninfected adults and nymphs after a 1-day (1D) or 3-day (3D) starvation period, as determined by stereomicroscopy.

**Fig 2:**
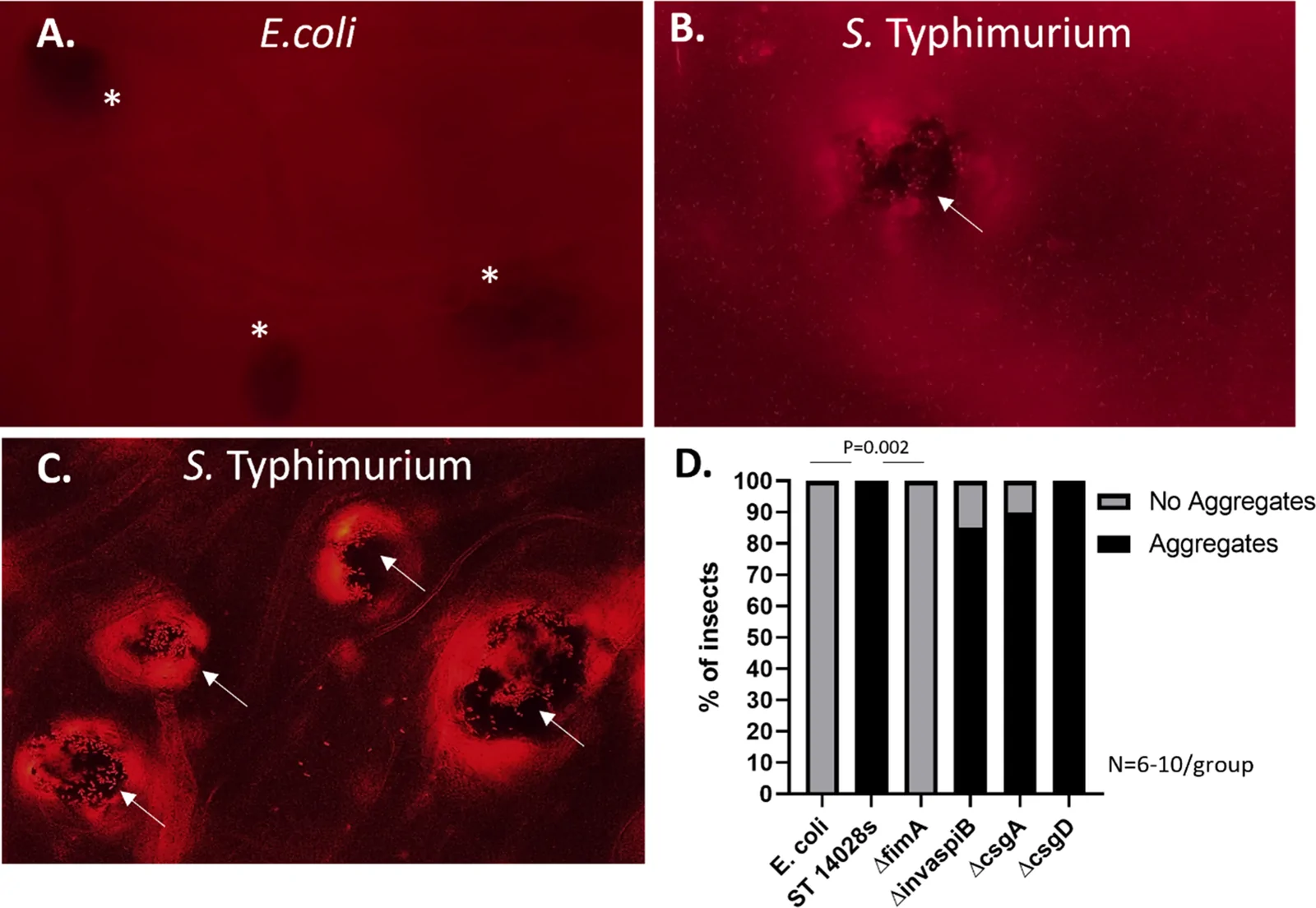
Aggregate formation by *S*. Typhimurium on melanized deposits is dependent on *fimA* but not c*sgA* or cs*gD*. (**A**) Melanized deposits with no bacterial aggregates (asterisks) 3 h post-infection with mCherry-expressing *E. coli*. (**B and C**) Melanized deposits with large aggregates (arrows) of mCherry-expressing *S*. Typhimurium 3 h post-infection. The image in panel C was processed with background subtraction and planktonic bacteria are also visible. (**D**) Prevalence of aggregate formation by *E. coli,* wild-type *S*. Typhimurium (14028s), and various mutant strains of *S*. Typhimurium, as determined by fluorescence microscopy 3 h post-infection. Data were analyzed using Fisher’s exact test.

When we dissect guts from uninfected colony derived cockroaches, we frequently observe melanized deposits in the foregut (Fig. 1A and B). Respectively, these were noted by stereomicroscopy in 4/12 nymphs examined after a 1-day starvation period, in 6/12 adult males examined after a 1-day starvation period, and in 8/12 adult males examined after a 3-day starvation period (Fig. 1C). The deposits are consistent in appearance with melanization produced as an immune response to bacteria and tissue damage that occurs in other insects both systemically (13, 30 – 32) and in the gut (33, 34). When adult male cockroaches were orally infected with wild-type *S*. Typhimurium, aggregates of bacteria were observed to form specifically around the melanized deposits in the foregut within the first 3 h of infection (Fig. 2B and C). This phenomenon was specific to *S*. Typhimurium and was seen in 100% of infected insects examined by fluorescence microscopy but did not occur when the cockroaches were infected with *Escherichia coli* (Fig. 2A and D, Fisher’s exact test, *P* = 0.002).

### Aggregate formation on melanized deposits by *S*. Typhimurium is dependent on *fimA* but not *csgA* or *csgD*

To examine the mechanism(s) behind formation of the aggregates, we orally infected cockroaches with 1 of 5 different strains of *S*. Typhimurium (wild-type, Δ*fimA*, Δ*invAspiB*, Δ*csgA*, and Δ*csgD*) and examined foreguts 3 h post-infection for the presence of bacterial aggregates around the melanized deposits (Fig. 2D). The deletion mutants were chosen to determine if the respective functions of the deleted genes in biofilm formation, adhesion, and host manipulation are required for aggregate formation around melanized deposits in the cockroach gut. The gene *fimA* encodes the major structural subunit of type 1 fimbriae, *invA* and *spiB* encode essential components of type III secretion systems 1 and 2, *csgD* is a major transcriptional regulator of biofilm formation, and *csgA* encodes the major curlin subunit, another critical component of biofilms. Interestingly, there was no significant difference between the proportion of guts displaying *S*. Typhimurium aggregates around melanized deposits in groups infected with either the wild-type strain, *ΔcsgA*, *ΔcsgD*, or *ΔinvAspiB*. However, there was a significant reduction in the proportion of guts displaying aggregates in the *ΔfimA*-infected group relative to the group infected with wild-type (Fig. 2D, Fisher’s exact test, *P* = 0.002). The reduction was stark, as the *ΔfimA* strain was never observed to form aggregates. This result indicates that several of the biofilm-associated genes we tested are not necessary for the formation of aggregates around melanized foregut deposits by *S*. Typhimurium. Furthermore, it establishes that the aggregates depend on *fimA* for formation.

### Melanized deposits in the cockroach foregut are induced by the bacterial microbiota and demonstrate properties consistent with melanin

**Fig 3:**
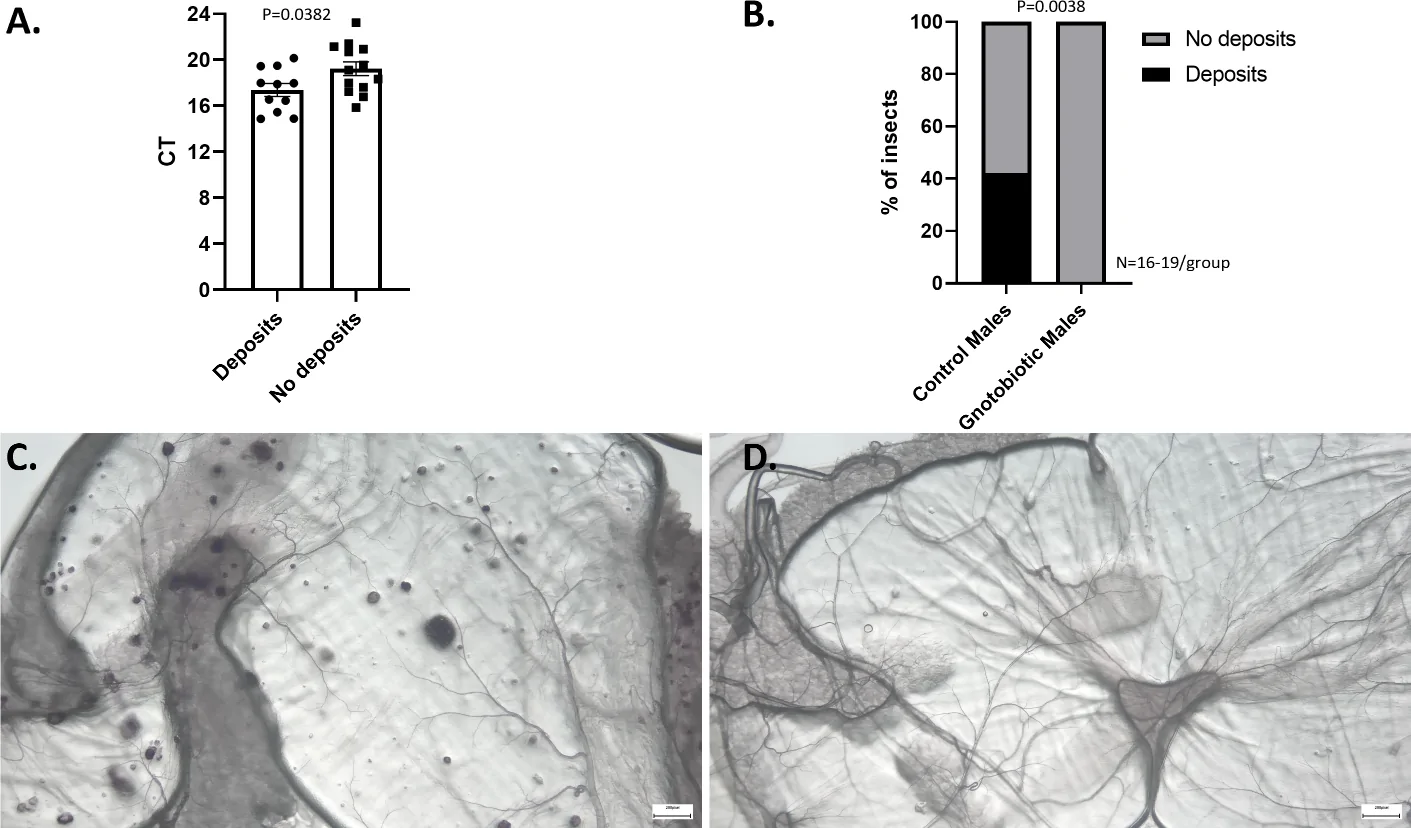
Melanized deposits in the cockroach gut are linked to the bacterial microbiota. (**A**) Bacterial density in guts from control (colony derived) cockroaches that exhibit or do not exhibit melanized deposits as determined by semi-quantitative PCR. Data were analyzed using a two-tailed parametric *t*-test. (**B**) Proportion of guts with melanized deposits in control and gnotobiotic adult males as determined by stereomicroscopy. Data were analyzed by Fisher’s exact test. (**C**) Representative micrograph of melanized deposits in the gut of a control (colony derived) cockroach with a normal gut microbiota. Note that the brightfield image from Fig. 1B, which is representative of the same experimental group, is used again in Fig. 3C for side-by-side comparison to the result shown in Fig. 3D. (**D**) Representative micrograph of a gut from a gnotobiotic cockroach without an environmentally acquired microbiota, which does not contain melanized deposits. Scale bars = 150 µm.

Based on their appearance, we hypothesized that the melanized deposits present in the cockroach foregut around which *S*. Typhimurium forms aggregates are composed of melanin produced as an immune response to commensal bacteria. We performed a series of four different experiments to test this hypothesis. First, we compared the density of the bacterial microbiota in guts from colony derived adult male cockroaches binned based on the presence or absence of melanized deposits in the foregut using a semi-quantitative PCR (qPCR) assay targeting a conserved region of the bacterial *16S rRNA* gene (Fig. 3A). In this assay, cockroach guts that had visible melanized deposits had an average cycle threshold (CT) value of 17.38. This was significantly lower than the average CT value of guts that did not have visible melanized deposits, which was 19.22 (*t*-test, *P* = 0.0382, Fig. 3A). This difference indicates a 3.58-fold increase in bacterial density in guts with melanized deposits relative to those without.

To further investigate the link between the gut microbiota and the presence of melanized deposits, we next used an established method to generate gnotobiotic cockroaches by rearing them in a sterile environment lacking environmental bacteria (35), which comprise the gut microbiota of *B. germanica* (36 – 38). We then examined the guts of these insects for the prevalence of melanized deposits in the foregut and compared this to the prevalence in guts of control (colony derived) cockroaches (Fig. 3B and D). Eight of nineteen control samples had melanized deposits in the gut, consistent with our initial observations (Fig. 1). On the other hand, 0/16 guts from gnotobiotic cockroaches had melanized deposits, which was a significantly lower proportion than was observed in the control group (Fisher’s exact test, *P* = 0.0038, Fig. 3B and D). This pattern shows that the presence of melanized deposits in the gut is associated with the gut microbiota and supports the qPCR data linking melanized deposits to a higher density of bacteria in the gut. Additionally, both results support the hypothesis that the melanized deposits may originate from an immune response to increased levels of commensal bacteria in the gut.

**Fig 4:**
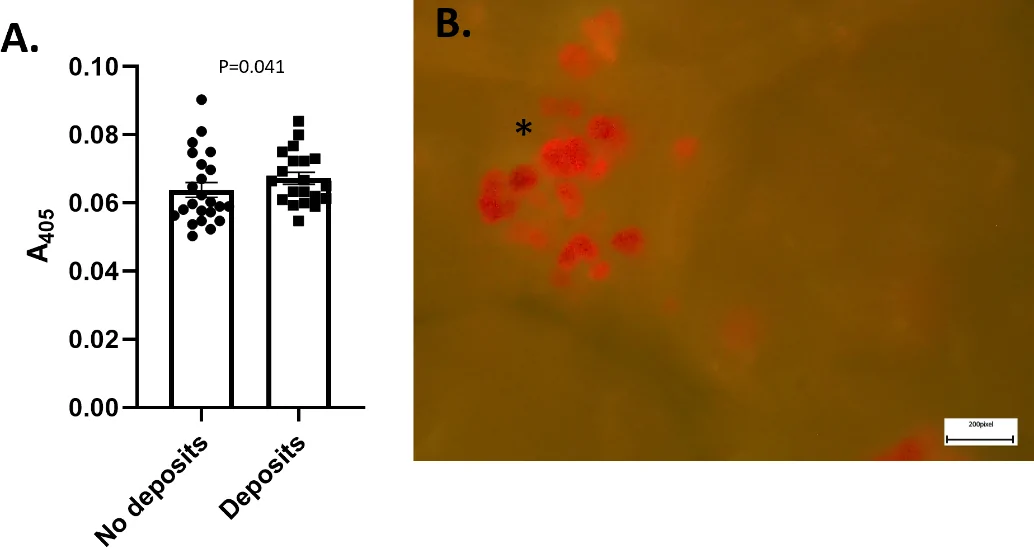
Melanized deposits in the cockroach gut exhibit properties consistent with melanin. (**A**) Absorbance at 405 nm in gut extracts from control (colony derived) cockroaches with and without melanized deposits. Data were analyzed by one-tailed non-parametric Mann–Whitney test. (**B**) Autofluorescence of melanized deposits (*) in the cockroach gut after fixation. Scale bar = 30 µm.

We lastly carried out two complementary experiments to determine if the deposits observed in the foregut are indeed composed of melanin. Melanin is known to strongly absorb light at 405 nm (39, 40). Direct absorbance measurements on dissolved gut tissues revealed higher absorbance at 405 nm in gut samples with melanized deposits relative to those without (Fig. 4A). This difference was small and may have been confounded by absorbance from other nitrophenol compounds in the gut but was nonetheless statistically significant (Mann–Whitney test, *P* = 0.041). Furthermore, when we fixed guts with melanized deposits in Carnoy’s solution overnight, the deposits exhibited red-orange autofluorescence (Fig. 4B), which has been previously reported for melanin deposits in the insect gut (41). Both of the above observations are strongly indicative that the melanized granules present in the cockroach gut contain melanin, though they are not entirely specific.

### Melanin deposits in the cockroach gut enhance *Salmonella* infection

**Fig 5:**
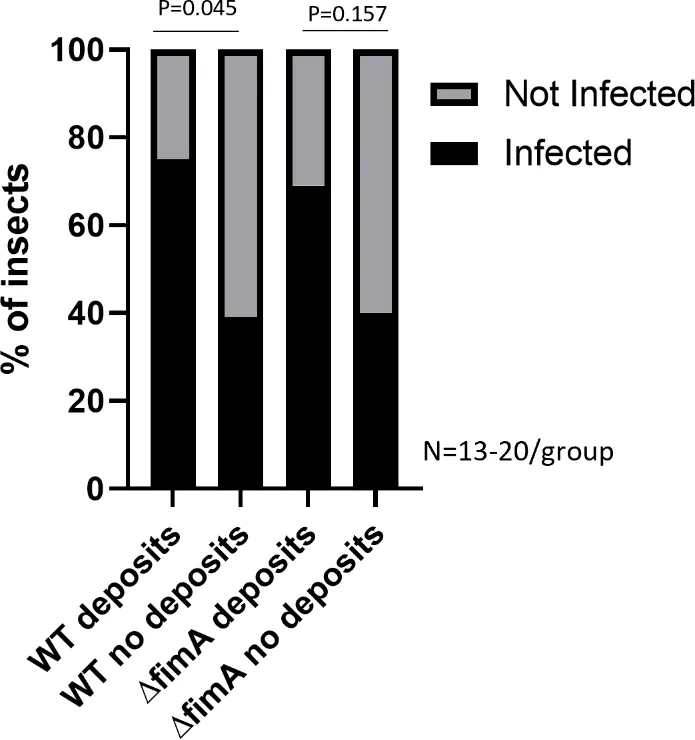
Melanin deposits in the cockroach gut enhance *S*. Typhimurium infection. Cockroaches were infected with wild-type (WT) or *fimA* deficient (Δ*fimA*) strains of *S*. Typhimurium. One day later, guts were dissected and sorted into groups of guts that contained melanin deposits and groups that did not, as determined by stereomicroscopy. The proportion of guts infected with *S*. Typhimurium was determined by plating gut homogenates on selective agar and scoring those with detectable colonies (limit of detection = 500 CFU). The data were analyzed by Fisher’s exact test.

To determine the effects of the microbiota-induced melanin deposits in infection of the cockroach vector by *S*. Typhimurium, we fed adult male cockroaches either the wild-type or Δ*fimA* strain. Twenty-four hours post-infection, we binned guts based on the presence or absence of melanin deposits and we compared the proportion of guts harboring detectable levels of *S*. Typhimurium (limit of detection = 500 CFU) by culture (Fig. 5). From these data, a significant decrease in the prevalence of infection with the wild-type strain was evident in guts without melanin deposits relative to those that had deposits (Fisher’s exact test, *P* = 0.045). Viable *S*. Typhimurium remained detectable 24 h post-infection in 75% of guts with melanized deposits, while only 39% of the guts without melanized deposits retained *S*. Typhimurium at the same time point. Furthermore, we did not see a significant difference in the proportion of guts infected with the Δ*fimA* strain between those that had melanin deposits and those that did not (Fisher’s exact test, *P* = 0.157), although a similar pattern to the wild-type strain was observed. These results generally indicate that the presence of microbiota-induced melanin deposits in the gut enhances infection of the vector. However, the observation of a similar trend for both the wild-type and Δ*fimA* strain suggests that the enhancement of infection by melanin deposits cannot be attributed solely to bacterial aggregation on these deposits. Rather, additional positive interactions between the microbiota, melanin deposits, and *S*. Typhimurium infection that are independent of *fimA*-mediated aggregation may exist.

## DISCUSSION

Our current and prior findings demonstrate that distinct populations of *S*. Typhimurium exist during infection of the cockroach gut (Fig. 2) (11). Alongside planktonic bacteria in the gut lumen, aggregated populations frequently form using small granular melanized deposits as substrates (Fig. 2B and C). Based on the new observations reported here, there appears to be some level of specificity to *S*. Typhimurium aggregation, as aggregates are not formed by *E. coli* in the cockroach gut (Fig. 2D). Furthermore, *S*. Typhimurium aggregates require type 1 fimbriae (*fimA*) to form but not curli fibers (*csgA*) or the master transcriptional regulator of biofilm formation (*csgD*) (Fig. 2D), revealing that the aggregates are not canonical biofilms. These findings extend the functional role of *fimA* in host colonization from mammals to an insect host, as *fimA* is involved in biofilm formation on gallstones in mice (28). However, the role of bacterial aggregation in colonization of the cockroach gut may not be restricted to *S*. Typhimurium, as it is still unclear if other types of fimbriae may enable other bacterial pathogens to behave similarly in the cockroach gut.

The melanized granules in the cockroach gut that act as substrates for *S*. Typhimurium aggregation show several properties consistent with melanin that are not entirely specific. These include their microscopic appearance, autofluorescence, and absorbance at 405 nm (Fig. 1 and 4). The formation of melanin deposits, or melanization, is a well-studied phenomenon that has been documented in the gut tissue of numerous insect species (32 – 34, 41, 42), including in the foregut (13, 42 – 44). Melanization occurs as an immune response to microbes as well as in response to tissue damage. In our work, melanin deposits in the cockroach gut were not age or starvation dependent (Fig. 1) but were correlated with gut bacterial density and were abrogated in gnotobiotic insects lacking a gut microbiota (Fig. 3). These results together strongly indicate that the gut melanization we observed is an immune response to the gut microbiota, but tissue damage may also play a role that cannot be ruled out by our data alone. An additional limitation of our data is that they do not identify the specific bacteria linked to melanization, which could be further explored through 16S rRNA profiling of the gut microbiota.

It has been previously shown that the German cockroach microbiota confers colonization resistance against ingested *E. coli* (35). However, effects of the microbiota on different ingested bacteria may vary and may also be context dependent. German cockroaches acquire a highly diverse gut microbiota horizontally from the environment via their diet and conspecific coprophagy (36 – 38, 45). As such, it is possible that the microbiota may have pleiotropic effects on infection through multiple mechanisms, simultaneously providing both positive and negative regulation. The revelation of aggregated and planktonic populations of *S*. Typhimurium further confounds understanding the microbiota’s role in infection, as distinct commensal species may differently affect the two populations.

Indeed, we show that melanin deposits, and by extension the gut microbiota, enhance *S*. Typhimurium infection of the cockroach gut (Fig. 5). One possible mechanism behind this observation is that melanin deposits act as substrates for *fimA-*mediated aggregation, and this is necessary for optimal infection. Nonetheless, in our infection assays, both the wild-type and Δ*fimA* mutant strains of *S*. Typhimurium showed similar deficiencies in colonization when melanin deposits were not present in the gut, although the deficiency was not statistically significant for the mutant strain. Thus, enhanced infection in guts with melanin deposits cannot be explained solely by bacterial aggregation on these substrates. The correlation between melanin deposits and a higher density of commensal bacteria in the gut points to additional positive interactions between some gut microbiota constituents and *S*. Typhimurium. For instance, some commensal bacteria that are linked to the formation of melanin deposits may enhance *S*. Typhimurium infection by producing beneficial metabolites, altering the microenvironment, or modulating immune responses to the pathogen in the cockroach gut (46 – 49).

**Fig 6:**
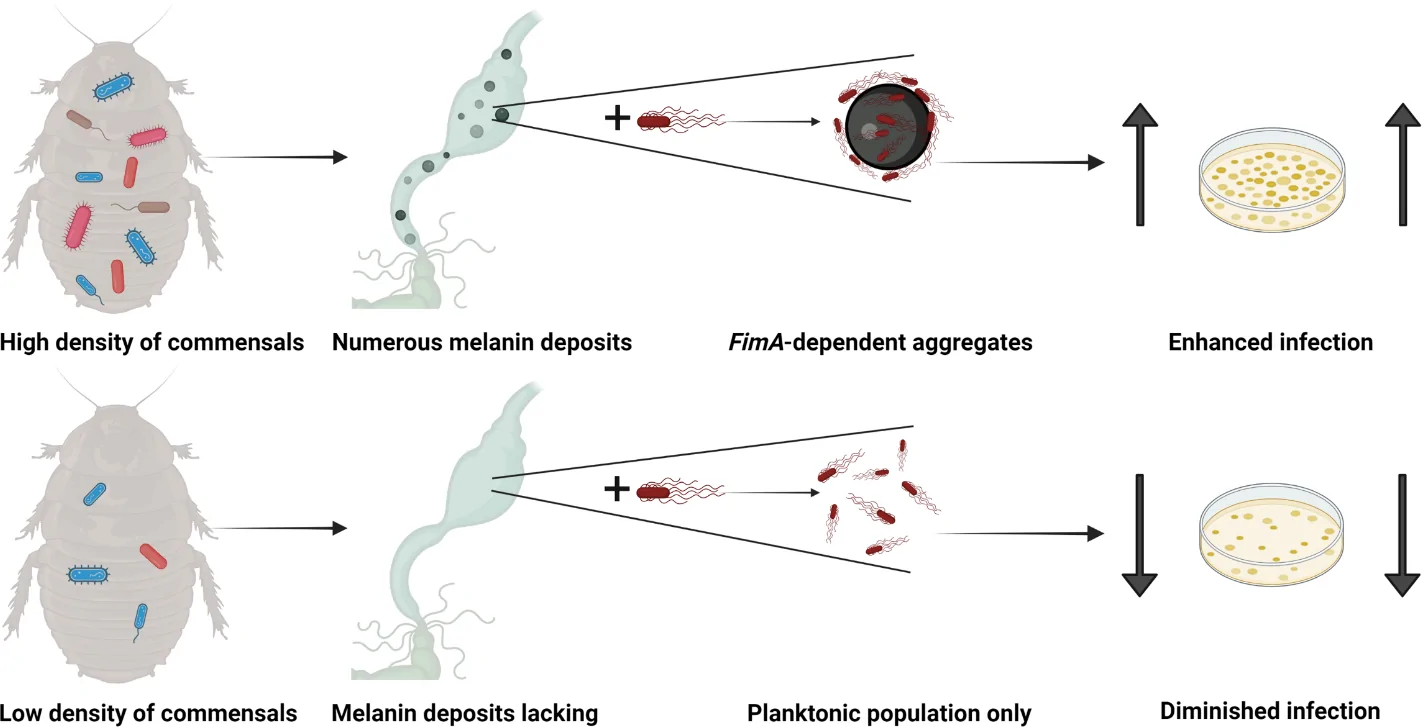
Summary of results. Higher densities of commensal bacteria in the cockroach gut correlate with the formation of melanized deposits, likely as an immune response. Upon entering the cockroach gut, *S*. Typhimurium forms aggregates on the surface of melanized deposits, a process which is dependent on *fimA*. In the absence of melanized deposits, lower infection rates are observed. Figure created with BioRender (license # DF2591D × 1N).

In summary, higher densities of commensal bacteria in the cockroach gut correlate with the formation of melanized deposits, likely as an immune response. Upon entering the cockroach gut, *S*. Typhimurium forms aggregates on the surface of these melanized deposits, a process that is dependent on *fimA*. In the absence of melanized deposits, lower infection rates are observed (Fig. 6). These findings demonstrate a novel mechanism by which *S*. Typhimurium interacts with various components of the cockroach gut environment to enhance infection of the vector and underscore the emerging but still underappreciated complexity of this insect–pathogen relationship. We emphasize that this mechanism likely does not operate in isolation. As discussed above, additional effects of different microbiota constituents on infection through mechanisms such as cooperation, competitive exclusion, and regulation of innate immunity should be considered and investigated to better understand the complex relationship between the cockroach gut, its microbiota, and *S*. Typhimurium infection. Moreover, *S*. Typhimurium can actively colonize several other insects, such as house flies and leafhoppers (50, 51), and it is of interest to determine the extent to which mechanisms are conserved across these hosts, as well as across other enteric pathogens that cockroaches may acquire in nature.

## MATERIALS AND METHODS

### Cockroach colonies

The American Cyanamid Orlando laboratory strain of *Blattella germanica* was used for all experiments, as in our previous work on bacterial infections (11, 12, 35). Cockroach colonies were maintained in plastic enclosures at 25 ± 1°C and 40%–45% relative humidity on a 12:12 (L:D) hour photoperiod. The colonies were provided dog chow (Purina, St. Louis, MO, USA) and tap water *ad libitum*, and were given egg carton harborages for shelter. Mostly adult males were used in experiments in order to preserve females and nymphs for colony propagation and minimize physiological variation due to gonadotropic and developmental cycles.

### Observation of melanized deposits in cockroach guts

After starvation of food and water for 1 or 3 d, whole guts were dissected from colony derived adult males or nymphs and observed through either a stereomicroscope (M165FC with DFC310 FX camera, Leica, Wetzlar, Germany) or compound microscope (Primo Star with Axiocam 208, Zeiss, Oberkochen, Germany) under brightfield. The guts were visually scored for the presence or absence of melanized deposits in the foregut.

### Observation of *S*. Typhimurium aggregates around melanized deposits in cockroach guts

*S*. Typhimurium was provisioned orally to groups of adult male cockroaches starved of food and water for 3 d to promote consistent experimental feeding as described in our previous work (11, 12). In summary, bacterial cultures were grown overnight in LB at 37°C, diluted to OD_600_ = 1, then provided to the cockroaches as a sole food source for 30 min. Insects that did not feed during the 30 min period were removed from enclosures and excluded from further analysis. *S*. Typhimurium strain 14028s expressing mCherry from the plasmid pFPV served as the wild-type control (26, 52), as we previously showed it forms aggregates in the cockroach gut (11). In addition, several deletion mutants of the 14028s strain transformed with pFPV-mCherry were tested. These were as follows: Δ*fimA*, deficient in the major structural subunit of type 1 fimbriae, Δ*invAspiB*, a type III secretion system 1/2 double mutant, Δ*csgD*, deficient in a major transcriptional regulator of biofilm formation, and Δ*csgA*, deficient in major curlin subunit, a critical component of biofilms. *Escherichia coli* strain DH10B harboring pPFV-mCherry was also included as a control in the experiments.

Three hours post-infection, guts were screened for detection of bacterial aggregates around existing melanized deposits. Whole guts were dissected and placed on microscope slides for observation using an Axioskop 2 fluorescence microscope with an Axiocam 208 camera (Zeiss). Observations were confined to the foregut (the primary location of melanized deposits). Multiple planes were observed to confirm observed bacteria were coating the deposits as opposed to merely being above or below them and representative images were taken. Guts were scored as positive or negative for bacterial aggregates around melanized deposits and data were analyzed using Fisher’s exact test to compare the prevalence of aggregate formation by the different strains.

### Quantitation of gut microbiota density by real-time PCR

A semi-quantitative real-time PCR assay (qPCR) was carried out as in our previous work (53) to determine if guts from colony derived cockroaches with melanized deposits and those without harbor different densities of commensal bacteria. DNA was isolated from whole guts using the DNeasy blood and tissue kit (Qiagen, Germantown, MD, USA) according to the manufacturer’s protocol. These guts were binned based on the presence or absence of melanized deposits as determined by stereomicroscopy after starvation for 3 d. qPCR was performed on a QuantStudio 3 instrument (Applied Biosystems, Waltham, MA, USA) using the PowerUp SYBR Green Master Mix (Applied Biosystems), 50 ng of DNA as template, and previously published primers targeting a conserved region of the bacterial 16S rRNA gene (331F/797R, F: 5′-TCCTACGGGAGGCAGCAGT-3′, R: 5′-GGACTACCAGGGTATCTAATCCTGTT-3′) (54). These primers cover 83.1% of bacterial taxa based on estimation using the SILVA TestPrime tool (54), allowing for simple simultaneous quantitation of diverse microbiota constituents, which would not be possible via culture. Amplification conditions were as follows: 95°C for 5 min, followed by 40 cycles of denaturation at 95°C for 15 s, annealing at 50°C for 20 s, and extension at 72°C for 30 s. Cycle threshold (CT) values were obtained for individual gut samples as a proxy for bacterial density and the data were analyzed by parametric two-tailed *t*-test as the assumption of normality was met.

### Analysis of gnotobiotic cockroaches

German cockroaches acquire their diverse gut microbiota horizontally from the environment via their diet and conspecific coprophagy (36 – 38, 45). To study the impact the microbiota has on the generation of melanized deposits in the gut, we developed gnotobiotic cockroaches which lack an environmentally acquired gut microbiota using a process established in our lab (35). The first step in the process involves gently removing and surface sterilizing mature oothecae from gravid females using 10% bleach and 70% ethanol. The sterilized oothecae and the nymphs derived from these are then maintained in a sterilized, air-tight container ventilated through a 0.2 µM filter with autoclaved water and dog chow (Purina). To verify our method, we periodically tested gnotobiotic cockroaches by culturing whole insect homogenates on LB agar without antibiotics to rule out contamination of the gnotobiotic colony. Once the gnotobiotic cockroaches molted into adults, these were screened for the presence of melanized deposits in the gut. To do so, we starved both gnotobiotic and control (colony derived) cockroaches of food and water for 3 d. After this, we dissected whole guts and scored them based on the presence or absence of melanized deposits in the foregut using stereomicroscopy as described above. Fisher’s exact test was used to compare gnotobiotic and colony derived groups.

### Quantitation of melanin in cockroach guts

To further investigate if the melanized deposits observed in the cockroach gut do in fact contain melanin, we carried out two additional experiments. In the first experiment, we adapted a protocol to quantify melanin concentration in cells based on absorbance of light at 405 nm (39, 40). Colony derived adult male cockroaches were starved for 3 d. Whole guts were dissected and scored based on the presence or absence of melanized deposits in the foregut by stereomicroscopy. The guts were then homogenized using a pestle in 500 µL of 1 N NaOH/10% DMSO solution. One hundred microliters of each sample in triplicate was loaded into a 96-well plate and absorbance at 405 nm was measured on a plate reader. Synthetic melanin (Sigma Aldrich, St. Louis, MO, USA) dissolved in the same solution was used as a positive control. A non-parametric one-tailed Mann–Whitney test was used to compare absorbance values in guts with or without melanin deposits as the assumption of normality was not met.

In a second experiment, we followed a process for detecting melanin autofluorescence by fixation in Carnoy’s solution (41). A solution of ethanol-chloroform-acetic acid, 6:3:1 [vol/vol] was used to fix three individual whole guts from colony derived males overnight. Autofluorescence was then observed using an Axioskop 2 fluorescence microscope with an Axiocam 208 camera (Zeiss).

### Quantitation of *Salmonella* infections

To examine the impact of melanin deposits in the gut on *S*. Typhimurium colonization, groups of adult male cockroaches were orally provisioned either wild-type or Δ*fimA S*. Typhimurium as described above. One day post-infection, insects were collected, whole guts were dissected, and the presence of foregut melanin deposits was noted by stereomicroscopy. Then, the whole guts were homogenized in sterile PBS and plated on LB agar containing 100 µg/mL ampicillin to quantify *S*. Typhimurium. Fisher’s exact test was used to compare the proportion of guts with detectable *S*. Typhimurium in the presence or absence of melanin deposits.

## Data Availability

All relevant data are contained within the manuscript.

## References

[1] Gore JC , Schal C . 2007. Cockroach allergen biology and mitigation in the indoor environment. Annu Rev Entomol 52:439–463. doi:10.1146/annurev.ento.52.110405.091313 17163801

[2] Nasirian H . 2019. Contamination of cockroaches (Insecta: Blattaria) by medically important Bacteriae: a systematic review and meta-analysis. J Med Entomol 56:1534–1554. doi:10.1093/jme/tjz095 31219601

[3] Graffar M , Mertens S . 1950. Rôle of Blatta in transmission of salmonellosis. Ann Inst Pasteur (Paris) 79:654–660. 14799998

[4] Donkor ES . 2020. Cockroaches and food-borne pathogens. Environ Health Insights 14:1178630220913365. doi:10.1177/1178630220913365 32425541 PMC7218330

[5] Burgess NR , Chetwyn KN . 1981. Association of cockroaches with an outbreak of dysentery. Trans R Soc Trop Med Hyg 75:332–333. doi:10.1016/0035-9203(81)90355-2 7029807

[6] Tarshis IB . 1962. The cockroach--a new suspect in the spread of infectious hepatitis. Am J Trop Med Hyg 11:705–711. doi:10.4269/ajtmh.1962.11.705 13993369

[7] Ash N , Greenberg B . 1980. Vector potential of the German cockroach (Dictyoptera: Blattellidae) in dissemination of Salmonella enteritidis serotype Typhimurium. J Med Entomol 17:417–423. doi:10.1093/jmedent/17.5.417 6999154

[8] Klowden MJ , Greenberg B . 1976. Salmonella in the American cockroach: evaluation of vector potential through dosed feeding experiments. J Hyg (Lond) 77:105–111. doi:10.1017/s0022172400055571 789761 PMC2129709

[9] Kopanic RJ , Sheldon BW , Wright CG . 1994. Cockroaches as vectors of Salmonella: laboratory and field trials. J Food Prot 57:125–135. doi:10.4315/0362-028X-57.2.125 31113148

[10] Zurek L , Schal C . 2004. Evaluation of the German cockroach (Blattella germanica) as a vector for verotoxigenic Escherichia coli F18 in confined swine production. Vet Microbiol 101:263–267. doi:10.1016/j.vetmic.2004.04.011 15261999

[11] Turner M , Peta V , Pietri JE . 2022. New insight into the relationship between Salmonella typhimurium and the German cockroach suggests active mechanisms of vector-borne transmission. Res Microbiol 173:103920. doi:10.1016/j.resmic.2021.103920 34954364

[12] Turner M , Pietri JE . 2022. Antimicrobial peptide expression in the cockroach gut during enterobacterial infection is specific and influenced by type III secretion. Biol Open 11:bio059414. doi:10.1242/bio.059414 35611712 PMC9167622

[13] Whitten MMA , Coates CJ . 2017. Re-evaluation of insect melanogenesis research: views from the dark side. Pigment Cell Melanoma Res 30:386–401. doi:10.1111/pcmr.12590 28378380

[14] Desai SK , Kenney LJ . 2019. Switching lifestyles is an in vivo adaptive strategy of bacterial pathogens. Front Cell Infect Microbiol 9:421. doi:10.3389/fcimb.2019.00421 31921700 PMC6917575

[15] Simm R , Ahmad I , Rhen M , Le Guyon S , Römling U . 2014. Regulation of biofilm formation in Salmonella enterica serovar Typhimurium. Future Microbiol 9:1261–1282. doi:10.2217/fmb.14.88 25437188

[16] Harrell JE , Hahn MM , D’Souza SJ , Vasicek EM , Sandala JL , Gunn JS , McLachlan JB . 2020. Salmonella biofilm formation chronic infection, and immunity within the intestine and hepatobiliary tract. Front Cell Infect Microbiol 10:624622. doi:10.3389/fcimb.2020.624622 33604308 PMC7885405

[17] MacKenzie KD , Palmer MB , Köster WL , White AP . 2017. Examining the link between biofilm formation and the ability of pathogenic Salmonella strains to colonize multiple host species. Front Vet Sci 4:138. doi:10.3389/fvets.2017.00138 29159172 PMC5581909

[18] Gerstel U , Park C , Römling U . 2003. Complex regulation of csgD promoter activity by global regulatory proteins. Mol Microbiol 49:639–654. doi:10.1046/j.1365-2958.2003.03594.x 12864849

[19] Ledeboer NA , Jones BD . 2005. Exopolysaccharide sugars contribute to biofilm formation by Salmonella enterica serovar Typhimurium on HEp-2 cells and chicken intestinal epithelium. J Bacteriol 187:3214–3226. doi:10.1128/JB.187.9.3214-3226.2005 15838049 PMC1082824

[20] Zogaj X , Bokranz W , Nimtz M , Römling U . 2003. Production of cellulose and curli fimbriae by members of the family Enterobacteriaceae isolated from the human gastrointestinal tract. Infect Immun 71:4151–4158. doi:10.1128/IAI.71.7.4151-4158.2003 12819107 PMC162016

[21] Gibson DL , White AP , Snyder SD , Martin S , Heiss C , Azadi P , Surette M , Kay WW . 2006. Salmonella produces an O-antigen capsule regulated by AgfD and important for environmental persistence. J Bacteriol 188:7722–7730. doi:10.1128/JB.00809-06 17079680 PMC1636306

[22] Chen S , Feng Z , Sun H , Zhang R , Qin T , Peng D . 2021. Biofilm-formation-related genes csgD and bcsA promote the vertical transmission of Salmonella enteritidis in chicken. Front. Vet. Sci 7:625049. doi:10.3389/fvets.2020.625049 33521095 PMC7840958

[23] El Hag M , Feng Z , Su Y , Wang X , Yassin A , Chen S , Peng D , Liu X . 2017. Contribution of the csgA and bcsA genes to Salmonella enterica serovar Pullorum biofilm formation and virulence. Avian Pathol 46:541–547. doi:10.1080/03079457.2017.1324198 28470089

[24] Purdy AE , Watnick PI . 2011. Spatially selective colonization of the arthropod intestine through activation of Vibrio cholerae biofilm formation. Proc Natl Acad Sci U S A 108:19737–19742. doi:10.1073/pnas.1111530108 22106284 PMC3241763

[25] Desai SK , Winardhi RS , Periasamy S , Dykas MM , Jie Y , Kenney LJ . 2016. The horizontally-acquired response regulator SsrB drives a Salmonella lifestyle switch by relieving biofilm silencing. Elife 5:e10747. doi:10.7554/eLife.10747 26880544 PMC4769171

[26] Desai SK , Padmanabhan A , Harshe S , Zaidel-Bar R , Kenney LJ . 2019. Salmonella biofilms program innate immunity for persistence in Caenorhabditis elegans. Proc Natl Acad Sci U S A 116:12462–12467. doi:10.1073/pnas.1822018116 31160462 PMC6589656

[27] Kolenda R , Ugorski M , Grzymajlo K . 2019. Everything you always wanted to know about Salmonella type 1 fimbriae, but were afraid to ask. Front Microbiol 10:1017. doi:10.3389/fmicb.2019.01017 31139165 PMC6527747

[28] Gonzalez-Escobedo G , Gunn JS . 2013. Identification of Salmonella enterica serovar Typhimurium genes regulated during biofilm formation on cholesterol gallstone surfaces. Infect Immun 81:3770–3780. doi:10.1128/IAI.00647-13 23897604 PMC3811749

[29] Alshalchi S , Hayer SS , An R , Munoz-Aguayo J , Flores-Figueroa C , Nguyen R , Lauer D , Olsen K , Alvarez J , Boxrud D , Cardona C , Vidovic S . 2017. The possible influence of non-synonymous point mutations within the fimA adhesin of non-typhoidal Salmonella (NTS) isolates in the process of host adaptation. Front Microbiol 8:2030. doi:10.3389/fmicb.2017.02030 29089942 PMC5651078

[30] Ayres JS , Schneider DS . 2008. A signaling protease required for melanization in Drosophila affects resistance and tolerance of infections. PLoS Biol 6:2764–2773. doi:10.1371/journal.pbio.0060305 19071960 PMC2596860

[31] Dudzic JP , Kondo S , Ueda R , Bergman CM , Lemaitre B . 2015. Drosophila innate immunity: regional and functional specialization of prophenoloxidases. BMC Biol 13:81. doi:10.1186/s12915-015-0193-6 26437768 PMC4595066

[32] Vodovar N , Vinals M , Liehl P , Basset A , Degrouard J , Spellman P , Boccard F , Lemaitre B . 2005. Drosophila host defense after oral infection by an entomopathogenic Pseudomonas species. Proc Natl Acad Sci U S A 102:11414–11419. doi:10.1073/pnas.0502240102 16061818 PMC1183552

[33] Chen J , Xie C , Tian L , Hong L , Wu X , Han J . 2010. Participation of the p38 pathway in Drosophila host defense against pathogenic bacteria and fungi. Proc Natl Acad Sci U S A 107:20774–20779. doi:10.1073/pnas.1009223107 21076039 PMC2996427

[34] Seisenbacher G , Hafen E , Stocker H . 2011. MK2-dependent p38b signalling protects Drosophila hindgut enterocytes against JNK-induced apoptosis under chronic stress. PLoS Genet 7:e1002168. doi:10.1371/journal.pgen.1002168 21829386 PMC3150449

[35] Ray R , Potts R , Pietri JE . 2020. The persistence of Escherichia coli infection in German cockroaches (Blattodea: Blattellidae) varies between host developmental stages and is influenced by the gut microbiota. J Med Entomol 57:1964–1971. doi:10.1093/jme/tjaa108 32516418

[36] Domínguez-Santos R , Pérez-Cobas AE , Artacho A , Castro JA , Talón I , Moya A , García-Ferris C , Latorre A . 2020. Unraveling assemblage, functions and stability of the gut microbiota of Blattella germanica by antibiotic treatment. Front Microbiol 11:487. doi:10.3389/fmicb.2020.00487 32269557 PMC7109288

[37] Rosas T , García-Ferris C , Domínguez-Santos R , Llop P , Latorre A , Moya A . 2018. Rifampicin treatment of Blattella germanica evidences a fecal transmission route of their gut microbiota. FEMS Microbiol Ecol 94:fiy002. doi:10.1093/femsec/fiy002 29325007

[38] Pérez-Cobas AE , Maiques E , Angelova A , Carrasco P , Moya A , Latorre A . 2015. Diet shapes the gut microbiota of the omnivorous cockroach Blattella germanica. FEMS Microbiol Ecol 91:fiv022. doi:10.1093/femsec/fiv022 25764470

[39] Zhou S , Sakamoto K , Hou L . 2020. Citric acid promoted melanin synthesis in B16F10 mouse melanoma cells, but inhibited it in human epidermal melanocytes and HMV-II melanoma cells via the GSK3β/β-catenin signaling pathway. PLoS ONE 15:e0243565. doi:10.1371/journal.pone.0243565 33332393 PMC7746170

[40] Netcharoensirisuk P , Abrahamian C , Tang R , Chen C-C , Rosato AS , Beyers W , Chao Y-K , Filippini A , Di Pietro S , Bartel K , Biel M , Vollmar AM , Umehara K , De-Eknamkul W , Grimm C . 2021. Flavonoids increase melanin production and reduce proliferation, migration and invasion of melanoma cells by blocking endolysosomal/melanosomal TPC2. Sci Rep 11:8515. doi:10.1038/s41598-021-88196-6 33875769 PMC8055690

[41] Engel P , Bartlett KD , Moran NA . 2015. The bacterium Frischella perrara causes scab formation in the gut of its honeybee host. mBio 6:e00193–15. doi:10.1128/mBio.00193-15 25991680 PMC4442143

[42] Shao Q , Yang B , Xu Q , Li X , Lu Z , Wang C , Huang Y , Söderhäll K , Ling E . 2012. Hindgut innate immunity and regulation of fecal microbiota through melanization in insects. J Biol Chem 287:14270–14279. doi:10.1074/jbc.M112.354548 22375003 PMC3340165

[43] Pulpitel T , Pernice M , Simpson SJ , Ponton F . 2015. Tissue-specific immune gene expression in the migratory locust. Insects 6:368–380. doi:10.3390/insects6020368 26463191 PMC4553485

[44] Wu K , Zhang J , Zhang Q , Zhu S , Shao Q , Clark KD , Liu Y , Ling E . 2015. Plant phenolics are detoxified by prophenoloxidase in the insect gut. Sci Rep 5:16823. doi:10.1038/srep16823 26592948 PMC4655367

[45] Kakumanu ML , Maritz JM , Carlton JM , Schal C . 2018. Overlapping community compositions of gut and fecal microbiomes in lab-reared and field-collected German cockroaches. Appl Environ Microbiol 84:e01037-18. doi:10.1128/AEM.01037-18 29959246 PMC6102980

[46] Thiennimitr P , Winter SE , Bäumler AJ . 2012. Salmonella, the host and its microbiota. Curr Opin Microbiol 15:108–114. doi:10.1016/j.mib.2011.10.002 22030447 PMC3265626

[47] Gillis CC , Hughes ER , Spiga L , Winter MG , Zhu W , Furtado de Carvalho T , Chanin RB , Behrendt CL , Hooper LV , Santos RL , Winter SE . 2018. Dysbiosis-associated change in host metabolism generates lactate to support Salmonella growth. Cell Host Microbe 23:570. doi:10.1016/j.chom.2018.03.013 PMC5907491 29649446

[48] Spiga L , Winter MG , Furtado de Carvalho T , Zhu W , Hughes ER , Gillis CC , Behrendt CL , Kim J , Chessa D , Andrews-Polymenis HL , Beiting DP , Santos RL , Hooper LV , Winter SE . 2017. An oxidative central metabolism enables Salmonella to utilize microbiota-derived succinate. Cell Host Microbe 22:291–301. doi:10.1016/j.chom.2017.07.018 28844888 PMC5599368

[49] Yoo W , Choi J , Park B , Byndloss MX , Ryu S . 2021. A nitrogen metabolic enzyme provides Salmonella fitness advantage by promoting utilization of microbiota-derived carbon source. ACS Infect Dis 7:1208–1220. doi:10.1021/acsinfecdis.0c00836 33853321 PMC8603301

[50] Chifanzwa R , Nayduch D . 2018. Dose-dependent effects on replication and persistence of Salmonella enterica serovar Typhimurium in house flies (Diptera: Muscidae). J Med Entomol 55:225–229. doi:10.1093/jme/tjx179 29029218 PMC5850332

[51] Dundore-Arias JP , Groves RL , Barak JD . 2015. Influence of prgH on the persistence of ingested Salmonella enterica in the leafhopper Macrosteles quadrilineatus. Appl Environ Microbiol 81:6345–6354. doi:10.1128/AEM.01464-15 26150468 PMC4542225

[52] Drecktrah D , Levine-Wilkinson S , Dam T , Winfree S , Knodler LA , Schroer TA , Steele-Mortimer O . 2008. Dynamic behavior of Salmonella-induced membrane tubules in epithelial cells. Traffic 9:2117–2129. doi:10.1111/j.1600-0854.2008.00830.x 18785994 PMC2682622

[53] Zha C , Turner M , Ray R , Liang D , Pietri JE . 2023. Effects of copper and zinc oxide nanoparticles on German cockroach development, indoxacarb resistance, and bacterial load. Pest Manag Sci 79:2944–2950. doi:10.1002/ps.7472 36966487 PMC10330183

[54] Jian C , Luukkonen P , Yki-Järvinen H , Salonen A , Korpela K . 2020. Quantitative PCR provides a simple and accessible method for quantitative microbiota profiling. PLoS One 15:e0227285. doi:10.1371/journal.pone.0227285 31940382 PMC6961887

